# Outcomes of patients with acute respiratory failure on veno-venous extracorporeal membrane oxygenation requiring additional circulatory support by veno-venoarterial extracorporeal membrane oxygenation

**DOI:** 10.3389/fmed.2022.1000084

**Published:** 2022-09-23

**Authors:** Rolf Erlebach, Lennart C. Wild, Benjamin Seeliger, Ann-Kathrin Rath, Rea Andermatt, Daniel A. Hofmaenner, Jens-Christian Schewe, Christoph C. Ganter, Mattia Müller, Christian Putensen, Ruslan Natanov, Christian Kühn, Johann Bauersachs, Tobias Welte, Marius M. Hoeper, Pedro D. Wendel-Garcia, Sascha David, Christian Bode, Klaus Stahl

**Affiliations:** ^1^Institute of Intensive Care Medicine, University Hospital Zurich, Zurich, Switzerland; ^2^Department of Anaesthesiology and Intensive Care Medicine, University Hospital Bonn, Bonn, Germany; ^3^Department of Respiratory Medicine and German Centre of Lung Research (DZL), Hannover Medical School, Hanover, Germany; ^4^Department of Gastroenterology, Hepatology and Endocrinology, Hannover Medical School, Hanover, Germany; ^5^Department of Cardiothoracic, Transplant and Vascular Surgery, Hannover Medical School, Hanover, Germany; ^6^German Research Foundation (DFG), Clinical Research Group (KFO 311): “(Pre)terminal Heart and Lung Failure: Unloading and Repair”, Germany; ^7^Department of Cardiology and Angiology, Hannover Medical School, Hanover, Germany

**Keywords:** extracorporeal life support (ECLS), triple cannulation, acute respiratory distress syndrome, sequential organ failure assessment (SOFA) score, vasoactive inotropic score, shock, survival analysis

## Abstract

**Objective:**

Veno-venous (V-V) extracorporeal membrane oxygenation (ECMO) is increasingly used to support patients with severe acute respiratory distress syndrome (ARDS). In case of additional cardio-circulatory failure, some experienced centers upgrade the V-V ECMO with an additional arterial return cannula (termed V-VA ECMO). Here we analyzed short- and long-term outcome together with potential predictors of mortality.

**Design:**

Multicenter, retrospective analysis between January 2008 and September 2021.

**Setting:**

Three tertiary care ECMO centers in Germany (Hannover, Bonn) and Switzerland (Zurich).

**Patients:**

Seventy-three V-V ECMO patients with ARDS and additional acute cardio-circulatory deterioration required an upgrade to V-VA ECMO were included in this study.

**Measurements and main results:**

Fifty-three patients required an upgrade from V-V to V-VA and 20 patients were directly triple cannulated. Median (Interquartile Range) age was 49 (28–57) years and SOFA score was 14 (12–17) at V-VA ECMO upgrade. Vasoactive-inotropic score decreased from 53 (12–123) at V-VA ECMO upgrade to 9 (3–37) after 24 h of V-VA ECMO support. Weaning from V-VA and V-V ECMO was successful in 47 (64%) and 40 (55%) patients, respectively. Duration of ECMO support was 12 (6–22) days and ICU length of stay was 32 (16–46) days. Overall ICU mortality was 48% and hospital mortality 51%. Two additional patients died after hospital discharge while the remaining patients survived up to two years (with six patients being lost to follow-up). The vast majority of patients was free from higher degree persistent organ dysfunction at follow-up. A SOFA score > 14 and higher lactate concentrations at the day of V-VA upgrade were independent predictors of mortality in the multivariate regression analysis.

**Conclusion:**

In this analysis, the use of V-VA ECMO in patients with ARDS and concomitant cardiocirculatory failure was associated with a hospital survival of about 50%, and most of these patients survived up to 2 years. A SOFA score > 14 and elevated lactate levels at the day of V-VA upgrade predict unfavorable outcome.

## Introduction

Extracorporeal membrane oxygenation (ECMO) has become an integral part in supporting patients with severe acute respiratory distress syndrome (ARDS) at specialized referral centers, due to the results of the CESAR trial and affected by the pandemics of H1N1 in 2009 and SARS-CoV-2 in 2019–2022 (1–3), despite controversial results from the randomized EOLIA trial (4). A veno-venous (V-V) cannulation technique is primarily employed to correct life-threatening hypoxemia and/or hypercapnia and to enable lung protective ventilation strategies (5). In cases of additive refractory cardio-circulatory deterioration, an upgrade of the V-V-system using an additional arterial return cannula (termed V-VA ECMO) to retain sufficient organ perfusion has been used by experienced centers (6). In such a triple cannulation set-up, V-VA ECMO provides both respiratory and hemodynamic support potentially representing a therapeutic option for patients with ARDS who develop secondary severe hemodynamic impairment (or heart failure). However, the literature of ARDS patients with secondary shock supported by V-VA ECMO is scarce and confined to case reports (7–10) and small series (11–13). Moreover, patient populations were heterogeneous, including both primary cardiogenic shock patients (starting with V-A ECMO) who were later upgraded with an additional venous cannula for treatment of respiratory failure (8, 12), as well as patients with primary ARDS (starting on V-V ECMO) who were later upgraded to V-VA for treatment of secondary cardio-circulatory failure (10, 13). Heterogeneity in cannulation sequences makes conclusions about the outcomes of patients with ARDS that subsequently require an arterial cannulation, upgrade difficult. Additionally, no data exist concerning long-term survival of these patients beyond the period of critical care or hospital stay and the extent of chronic organ failure in survivors is unknown.

This retrospective study from three ECMO referral centers aimed at describing the short and long-term outcomes of a cohort of patients with predominant ARDS receiving V-V ECMO support who required an upgrade to V-VA ECMO because of additional cardio-circulatory failure. Additionally, factors associated with poor outcome of V-VA ECMO support strategy were analyzed.

## Materials and methods

### Design and study population

In this retrospective observational cohort study, we aimed to describe characteristics and outcome of patients with ARDS and additional acute cardio-circulatory failure under V-VA ECMO support. Data were collected from the clinical information system by the local study team of two centers in Germany (Hannover Medical School, University Hospital Bonn) and one center in Switzerland (University Hospital Zurich). Inclusion criteria were ARDS with V-V ECMO support and upgrade to V-VA ECMO or direct V-VA ECMO implantation to simultaneously treat primary respiratory failure and secondary cardio-circulatory deterioration during the period from January 2008 to September 2021. In the contributing centers an escalation from V-V to V-VA ECMO is considered in refractory shock after optimization of conventional respiratory and hemodynamic support. Patients with primary cardiac failure requiring V-A ECMO therapy that later developed respiratory failure and required additional venous cannulation (e.g., upgrade from V-A ECMO to V-AV ECMO) were excluded from this analyses. The study was approved by the institutional review boards at all sites (Ethikkommission Hannover Medical School: #9720 BO K 2021, 2021/04/21; Kantonale Ethikkommission Zürich: ZH 2021-01804, 2021/10/08; Ethikkommission University Hospital Bonn: #488/21, 2021/05/07). Informed consent was waived by the regulatory body for all patients at both sites in Germany and for patient in Zurich before 2016 and later if death occurred before consent could be obtained. Consent has been obtained for all patients not falling under above conditions. All analyses performed involving human data were in accordance with the ethical standards of the institutional and national research committee of Switzerland and Germany and with the 1964 Helsinki Declaration and its latest amendments.

### Variables and definitions

Extracorporeal membrane oxygenation (ECMO) nomenclature based on the ELSO Maastricht Treaty for ECLS Nomenclature (14), where V-VA ECMO stands for an upgrade of V-V ECMO in patients with predominant ARDS with an additional arterial return cannula. Differential return blood flow of V-VA ECMO was regulated with gate clamps and additional flow monitors at the venous return cannula.

We collected demographic data, current illness leading to ECMO support and relevant comorbidities. Respiratory and hemodynamic parameters and the extent of organ support were analyzed at two time points – before V-V ECMO implantation and before V-VA ECMO upgrade. ECMO configuration and initial settings for V-V ECMO and V-VA ECMO were collected. The following outcome parameters were included: ECMO runtime, ICU and hospital length of stay, organ-specific outcomes (lung transplantation, long-term oxygen therapy, chronic kidney disease, congestive heart failure), mortality during ICU- and hospital stay and after one and two years. Additional, Vasoactive-inotropic score and serum lactate 24 h after V-VA ECMO upgrade was collected. If patients deceased in the first 24 h, the latest value before discontinuation of life-sustaining therapies was documented.

ARDS was defined according to the Berlin definition (15). ARDS was further classified as primary, when a direct lung insult was the most likely cause, or as secondary in case of an extra-pulmonary origin of ARDS. Primary ARDS was further divided into identified lung insults according to the RESP-score (16). The PRESERVE mortality risk score comprises pre-ECMO parameters that were shown to be correlated with mortality as a lower PRESERVE score is associated with a lower risk of death 6 months after ICU discharge (17). The Sequential Organ Failure Assessment (SOFA) score was used to assess the severity of organ dysfunction and to determine the predicted mortality risk (18). The Vasoactive-inotropic score (VIS) was used to quantify pharmacologic hemodynamic support by different inotropes and vasopressors and to compare it between groups (19).

We used a clinical definition of acute cardio-circulatory deterioration based on evidence of cardiac impairment on bed-side echocardiography or extended hemodynamic monitoring including cardiac output measurements, the degree of hemodynamic support, signs of impaired organ perfusion on clinical examination and laboratory parameters such as lactate levels and urine output. Left ventricular ejection fraction (LVEF) and right ventricular ejection fraction (RVEF) were semi-quantitatively categorized as good/sustained and reduced, respectively, because exact measurements were limited due to time-critical ECMO upgrade.

Comorbidities were extracted from the clinical information system. For immunosuppression we used the definition of the APACHE II Score (20, 21) and defined high-dose steroid therapy as prednisone-equivalent doses of ≥ 7.5 mg/day. Obesity was defined as body-mass index (BMI) of ≥ 30 kg/m^2^.

Intracranial hemorrhages were classified as minor when occasionally identified on routine cerebral imaging or as major when requiring neurosurgical intervention or resulting in any neurological deficit. Anemia requiring four or more red blood cell concentrates within 24 h after V-VA ECMO upgrade was chosen as a clinically relevant cut-off for bleeding complications.

### Statistical analysis

Comparison of variables between two time-points was performed using the Wilcoxon Signed Rank and Chi-Squared Test, as appropriate. A two-sided *p* < 0.05 was considered statistically significant. Comparison between variables at V-VA ECMO upgrade and 24 h follow-up was performed using the paired Wilcoxon Signed Rank Test. Clinically relevant population characteristics and characteristics at the time of V-VA ECMO implantation were stratified according to ICU-mortality and compared using Cox proportional-hazards model for 60-day ICU-mortality. Variables with a signification association in the univariate Cox-model were entered into the multivariate Cox-model. Ordinal variables (SOFA score) were further categorized into two groups with the cut-off chosen according to the receiver operating characteristic (ROC) curve and Youden Index. Proportional-hazards assumptions were checked visually and with Schoenfeld Individual Test. After model reduction method and input of interaction terms, variables were only retained if they were found to contribute to the model. Survival plots were generated for overall survival and 60-day survival stratified by variables in the multivariate Cox-model using the best cut-off chosen with ROC curve and Youden Index. Missing data are indicated in Supplementary Tables 1–4 of the Supplementary material.

## Results

### Population characteristics

In the three study centers, 73 patients met the inclusion criteria and were analyzed. In 53 (73%) patients V-V ECMO was upgraded to V-VA ECMO after a median of 48 (Interquartile Range, 8-120) hours. In 20 (27%) patients, primary V-VA ECMO support was applied due to simultaneous presence of respiratory and cardio-circulatory failure. Most common reason for respiratory failure was primary ARDS (*n* = 65, 89%), particularly bacterial pneumonia (*n* = 33, 47%). Table 1 summarizes the patient characteristics.

**TABLE 1:** Patient characteristics.

Variable	Overall (*N* = 73)
Age, years	49 (28–57)
Sex, female	24 (33)
Body-mass index, kg/m^2^	25 (22–30)
ARDS, primary	65 (89)
*Resp-Score diagnosis*	
Bacterial pneumonia	33 (47)
Viral pneumonia	11 (16)
Aspiration pneumonitis	7 (10)
Other acute respiratory diagnosis	10 (14)
Non-respiratory and chronic respiratory diagnoses	8 (11)
Trauma/burn	1 (1)
COVID-19	6 (8)
PRESERVE Score	4 (3–6)
Sepsis	58 (79)
*Comorbidities*	
Adipositas	19 (26)
COPD	10 (14)
Arterial hypertension	25 (34)
Coronary artery disease	5 (7)
Congestive heart failure	4 (5)
Diabetes mellitus	6 (8)
Chronic kidney disease	6 (8)
Immunosuppression	21 (29)
Solid organ transplantation	8 (11)

Values are expressed as n (%) or median (interquartile range). ARDS, acute respiratory distress syndrome; COPD, chronic obstructive pulmonary disease; COVID-19, Coronavirus disease 2019.

In those patients where echocardiographic data were available, reduced right ventricular systolic function was observed in 64% of patients before V-VA ECMO upgrade (25 out of 39 patients with available data). A trend toward higher vasopressor and inotropic doses was observed before V-VA ECMO upgrade, represented by a numerically higher median VIS of 27 (0–77) at V-V ECMO implantation and 53 (12–123) at V-VA ECMO upgrade (*p* = 0.054). Epinephrine was used significantly more frequently before V-VA ECMO (*n* = 18, 25%) than before V-V ECMO (*n* = 3, 6%) (*p* = 0.014). Eleven (15%) patients had undergone cardiopulmonary resuscitation before V-VA ECMO implantation. Clinical condition and organ support before V-V and V-VA ECMO are summarized and further stratified by initial V-VA ECMO implantation or later V-VA ECMO upgrade in Table 2.

**TABLE 2:** Clinical condition and organ support.

				Stratification at time of V-VA ECMO implantation/Upgrade		
Variables	Time of V-V ECMO implantation (*N* = 53)	Time of V-VA ECMO upgrade (*N* = 73)	*P*-value	Initial V-VA ECMO (*N* = 20)	V-V ECMO with later upgrade to V-VA (*N* = 53)	*P*-value
CPR before V-VA ECMO		11 (15)		5 (25)	6 (11)	0.276
Hospital admission to cannulation, days	6 (3-12)	11 (4–20)	0.027	10 (2–23)	11 (5–19)	0.719
ICU admission to cannulation, days	3 (1–8)	6 (2–13)	0.027	3 (1–6)	7 (3–13)	0.007
iMV to cannulation, days	1 (0–6)	3 (1–11)	0.011	1 (0–3)	6 (1–11)	0.006
SOFA score	13 (11–16)	14 (12–17)	0.179	12 (12–17)	14 (12–16)	0.129
*Respiratory support*			0.394			0.939
iMV	48 (92)	71 (97)		20 (100)	51 (96)	
NIV/HFOT	4 (8)	2 (3)		0 (0)	2 (4)	
PEEP, cmH_2_O	14 (11–16)	13 (10–16)	0.579	12 (10–14)	13 (10–16)	0.448
Minute ventilation, L/min	9.0 (7.0–11.0)	4.6 (2.7–8.1)	< 0.001	9.3(6.1–12.1)	4.2 (2.0–5.1)	0.001
Plateau pressure, cmH_2_O	30 (28–34)	28 (25–30)	0.046	30 (28–32)	28 (24–30)	0.057
SaO_2_,%	89 (82–92)	89 (79–93)	0.949	85 (72–92)	90 (83–93)	0.292
PaO_2_/FIO_2_, mmHg	71 (54–92)	67 (57–98)	0.876	62 (40–75)	69 (58–109)	0.074
PaCO_2_, mmHg	60 (51–68)	47 (41–55)	< 0.001	64 (56–71)	45 (39–49)	< 0.001
pH	7.23 (7.16-7.34)	7.31 (7.19–7.38)	0.054	7.20 (7.12–7.28)	7.34 (7.24–7.40)	0.003
Lactate, mmol/L	2.1 (1.3–3.7)	2.5 (1.6–5.9)	0.104	1.8 (1.3–2.5)	3.4 (1.9–6.9)	0.017
Inhalative nitric oxide	16 (32)	25 (36)	0.776	11 (58)	14 (28)	0.043
Norepinephrine	38 (76)	64 (89)	0.1	18 (90)	46 (88)	1.000
Norepinephrine dose, μg/kg/min	0.50 (0.23–0.89)	0.53 (0.19–1.08)	0.912	0.31 (0.16–0.67)	0.58 (0.20–1.25)	0.159
Epinephrine	3 (6)	18 (25)	0.014	5 (25)	13 (25)	1.000
Epinephrine dose, μg/kg/min	0.56 (0.30–0.78)	0.25 (0.08–0.64)	0.695	0.21 (0.17-1.03)	0.28 (0.08–0.57)	0.545
Dobutamine	7 (14)	22 (31)	0.066	5 (25)	17 (33)	0.727
Dobutamine dose, μg/kg/min	2.05 (1.77–4.69)	3.33 (2.04–4.15)	0.878	3.75 (3.75–4.29)	2.39 (1.40–3.75)	0.147
Vasoactive-inotropic score	27 (0–77)	53 (12–123)	0.054	30 (4–75)	57 (13–135)	0.304
*LVEF*			0.380			0.324
good/sustained	22 (88)	28 (76)		10 (91)	18 (69)	
reduced	3 (12)	9 (24)		1 (9)	8 (31)	
*RVEF*			0.001			0.250
good/sustained	20 (80)	14 (36)		6 (55)	8 (29)	
reduced	5 (20)	25 (64)		5 (45)	20 (71)	
Renal replacement therapy (n)	19 (37)	32 (44)	0.526	6 (30)	26 (49)	0.231

Left: Variables at V-V ECMO implantation and at V-VA ECMO implantation/upgrade. Right: Stratification at V-VA ECMO implantation by initial V-VA cannulation vs. initial V-V cannulation with later upgrade to V-VA. Values are expressed as n (%) or median (interquartile range). Doses of norepinephrine, epinephrine and dobutamine refer to the median dose of patients that received the drug. CPR, cardiopulmonary resuscitation; FIO_2_, fraction of inspired oxygen; HFOT, high-flow oxygen therapy; ICU, intensive care unit; iMV, invasive mechanical ventilation; LVEF, left ventricular ejection fraction (as estimated by echocardiography); NIV, non-invasive ventilation; PaCO_2_, partial pressure of carbon dioxide; PaO_2_, partial pressure of oxygen; PEEP, positive end-expiratory pressure; RVEF, right ventricular ejection fraction (as estimated by echocardiography); SaO_2_, arterial oxygen saturation; SOFA, Sequential Organ Failure Assessment.

### Extracorporeal membrane oxygenation configurations and complications

The femoral site for venous drainage (*n* = 66, 90%) and the jugular site for venous return (*n* = 63, 86%) was the most frequent configuration of V-V ECMO. Cannulation of both the femoral artery (*n* = 41, 56%) and the subclavian artery (*n* = 32, 44%) where used in V-VA ECMO upgrade. The most frequent complication following ECMO insertion was anemia requiring four or more red blood cell concentrates in 24 h (*n* = 38, 52%). ECMO configurations and complications are summarized in Table 3.

**TABLE 3:** Extracorporeal membrane oxygenation (ECMO) configuration, setting and complications.

Variable	Overall
**ECMO cannulation (*N* = 73)**	
*Venous drainage site*	
Femoral	66 (90)
Jugular	7 (10)
*Venous return site*	
Femoral	10 (14)
Jugular	63 (86)
*Arterial return site*	
Femoral	41 (56)
Subclavian	32 (44)
Antegrade leg perfusion cannula (% of patients with femoral cannulation)	28 (68)
**V-V ECMO settings (*N* = 53)**	
Pump speed, rpm	3000 (2885–3345)
Blood flow, L/min	4.0 (3.1–4.6)
Sweep gas flow, L/min	3 (2–4)
FsO_2_	1 (1–1)
**V-VA ECMO settings (*N* = 73)**	
Pump speed, rpm	3580 (3222–3938)
Total blood flow, L/min	5.0 (4.4–5.9)
Arterial blood flow, L/min	2 (2–3)
Sweep gas flow, L/min	6 (4–8)
**Complications of V-VA ECMO therapy (*N* = 73)**	
Complications during insertion	19 (26)
Complications during insertion requiring surgery	17 (23)
≥ 4 red blood cell concentrates/24 h	38 (52)
Major intracranial hemorrhage	5 (7)
Minor intracranial hemorrhage	5 (7)
Thromboembolic events	14 (19)
Leg ischemia	7 (10)
Other complications	13 (18)

Values are expressed as n (%) or median (interquartile range). FsO_2_: Sweep gas inlet oxygen fraction, major intracranial hemorrhage: requiring neurosurgical intervention or resulting in any neurological deficit, minor intracranial hemorrhage: occasionally identified on cerebral imaging, rpm: revolutions per minute.

### Outcome

**FIGURE 1:**
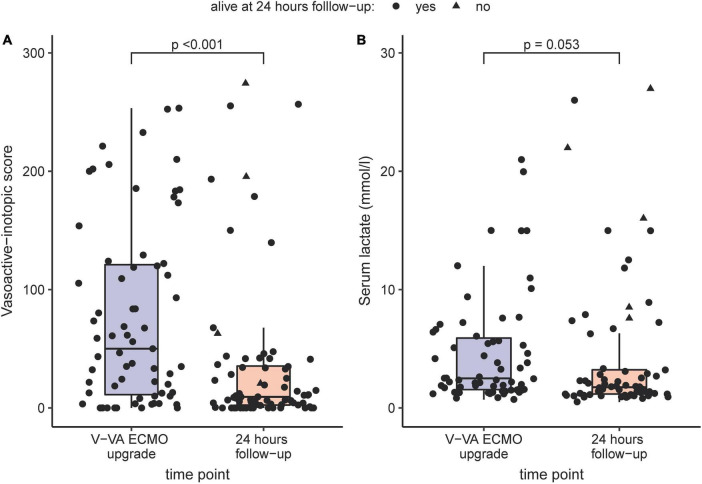
Comparison of Vasoactive-inotropic score **(A)** and serum lactate **(B)** before V-VA ECMO upgrade and after 24 h under V-VA ECMO support visualized as boxplots and scatterplots. If patients deceased in the first 24 h, the latest value before discontinuation of life-sustaining therapies was chosen.

VIS decreased significantly from 53 (12–123) at V-VA upgrade to 9 (3–37) after 24 h (*p* < 0.001). During the same time interval lactate levels decreased from 2.5 (1.6–5.9) to 1.8 (1.2–3.2) (*p* = 0.053). Both comparisons are visualized in Figure 1. V-VA ECMO and V-V ECMO was successful weaned in 47 (64%) and 40 (55%) patients, respectively. Thirty-five (48%) V-VA ECMO patients died during their ICU stay. Two patients (3%) died during the later hospital course. Of those patients, one died of pericardial tamponade and another patient died of recurrent respiratory failure due to progressive lung allograft dysfunction. After hospital discharge further two patients (3%) died during a two-year follow-up. Given that in six (8%) patients follow-up time was less than two years, an overall two year-mortality of 58% (39 of 67) was observed. Follow-up data and organ-specific outcomes are summarized in Table 4.

**TABLE 4:** Outcome and follow-up.

Variables	Overall (*N* = 73)
ECMO runtime, days	12 (6–22)
V-VA ECMO runtime, days	6 (3–9)
ICU length of stay, days	32 (16–46)
Hospital length of stay, days	44 (24–78)
ICU mortality	35 (48)
Hospital mortality	37 (51)
Lung Transplantation	12 (16)
Mortality at 1 year (*N* = 68)	39 (57)
Mortality at 2 years (*N* = 67)	39 (58)
**Organ specific outcome at 2 years (*N* = 28)**	
Long-term oxygen therapy	1 (4)
*Chronic kidney disease*	
KDIGO grade ≤3	25 (89)
KDIGO grade 4–5	1 (4)
unknown	2 (7)
*Congestive heart failure*	
NYHA stage ≤ 2	21 (75)
NYHA stage 3–4	0 (0)
unknown	7 (25)

Values are expressed as n (%) or median (interquartile range). ICU, intensive care unit; KDIGO, Kidney Disease: Improving Global Outcomes; NYHA, New York Heart Association.

### Predictors of intensive care unit mortality

**FIGURE 2:**
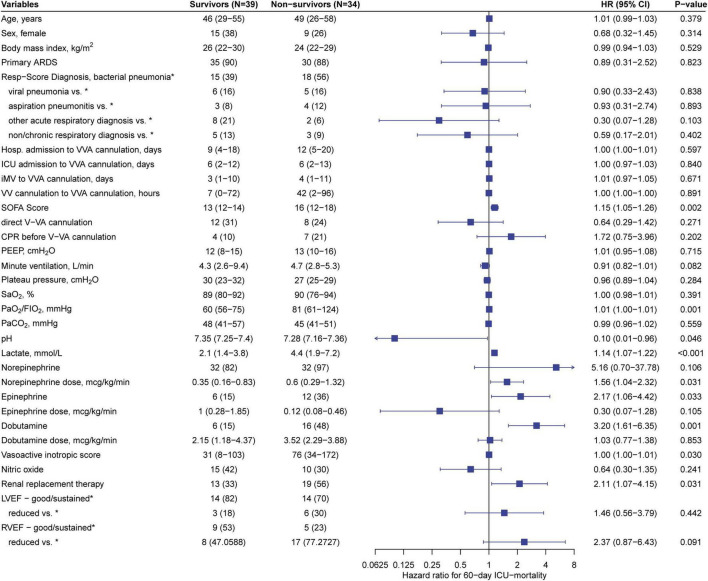
Stratification of predictive variables at veno-veno-arterial ECMO cannulation. *Left*: Predictive variables at the time of V-VA ECMO cannulation stratified in survivors and non-survivors according to 60-day ICU mortality. Values are expressed as n (%) or median (interquartile range). *Right*: Forest-plot and univariate cox regression for 60-day ICU-mortality. Values are expressed as Hazard ratio (HR) with 95% confidence interval (CI) and p-value. ARDS, acute respiratory distress syndrome; CPR, cardiopulmonary resuscitation; FIO_2_, fraction of inspired oxygen; Hosp., Hospital; ICU, intensive care unit; iMV, invasive mechanical ventilation; LVEF, left ventricular ejection fraction; PaCO_2_, partial pressure of carbon dioxide; PaO_2_, partial pressure of oxygen.

**FIGURE 3:**
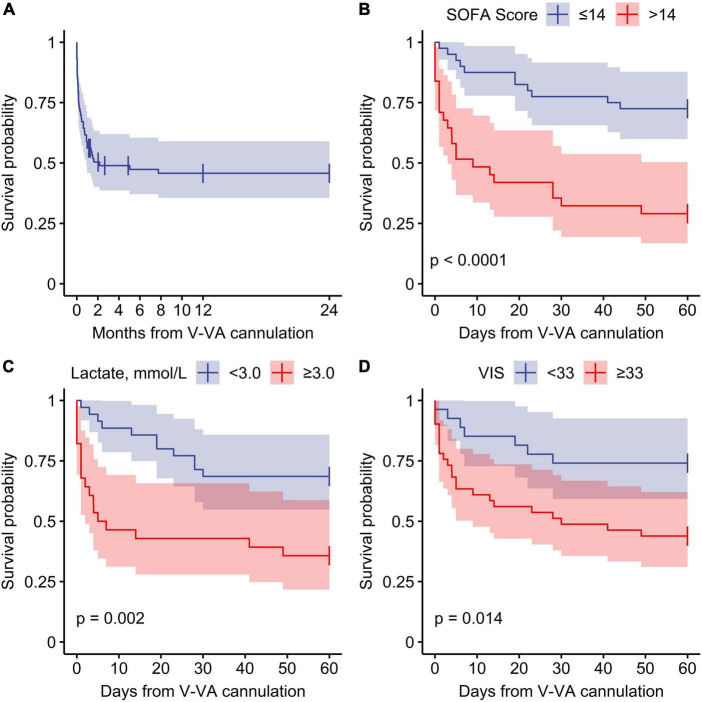
Survival function from veno-venoarterial ECMO cannulation. **(A)** Overall survival, **(B–D)** stratified survival function for 60-day ICU mortality. SOFA, Sequential Organ Failure Assessment; VIS, Vasoactive inotropic score.

Stratification of predictive variables at the time of V-VA ECMO implantation and results from Cox regression for 60-day ICU-mortality are shown in Figure 2. Of the variables that showed a significant association with 60-day ICU-mortality, five variables (SOFA score, lactate, VIS, renal replacement therapy and pH) were entered into the multivariate analysis. The PaO_2_/FIO_2_ ratio was excluded because it is not a reliable parameter for oxygen requirements under ECMO support. In the final multivariable model, SOFA score > 14 (Hazard ratio 4.28; 95% CI: 1.55–11.80, *p* = 0.005) and lactate level [(Hazard ratio 1.004; 95% CI: 1.000–1.008), *p* = 0.049] were significantly associated with 60-day ICU-mortality. Neither in-hospital nor 60-day nor 2-year survival was different between patients receiving initial V-VA cannulation and those receiving initial V-V cannulation with later V-VA upgrade (Supplementary Table 5). The results of the Cox proportional-hazards model are provided in Table 5. Survival plots stratified for these predictors are shown in Figure 3 and Supplementary Figure 1 (Supplementary material). Reduction in VIS 24 h after V-VA ECMO upgrade was significantly associated with improved survival (Supplementary Figure 2 and Supplementary Table 6).

**TABLE 5:** Cox proportional-hazards model for 60-day intensive care unit-mortality.

	Univariate			Multivariate		
Variables at V-VA ECMO implantation	HR	CI 95%	*P*-value	HR	CI 95%	*P*-value
SOFA score > 14	4.139	1.997–8.576	<0.001	4.275	1.548–11.805	0.005
Lactate, mmol/L	1.006	1.003–1.009	<0.001	1.004	1.000–1.008	0.049
VIS	1.004	1.000–1.007	0.034	1.001	0.997–1.006	0.632
Renal replacement therapy	2.107	1.069–4.153	0.031	0.830	0.328–2.101	0.694
pH	0.100	0.010–0.962	0.046	–	–	–

Values are expressed as Hazard ratio (HR) with 95% confidence interval (CI) and p-value. Variable were taken from time of V-VA-cannulation. SOFA: Sequential Organ Failure Assessment, VIS: Vasoactive-inotropic score.

## Discussion

In the present retrospective study, patients with predominant ARDS on V-V ECMO support who required additional V-VA ECMO support due to acute cardio-circulatory failure had an encouraging ICU survival rate of 52%. Two patients died during the later hospital course thereafter and only an additional two died in the two-years follow-up. Besides this unexpected high long-term survival only a minority of survivors suffered from relevant persistent organ dysfunction. A SOFA-score of more than 14 at the day of V-VA ECMO upgrade independently predicted an unfavorable outcome in these critically ill patients.

Previous studies and case series have found survival rates of patients with V-VA ECMO support ranging from 39 to 75% (11–13, 22–27). This wide range might be attributable to heterogeneity of the patient cohorts, including those with cardiogenic shock requiring initial V-A ECMO and later venous ECMO upgrade grouped together with ARDS patients on initial V-V ECMO support with a later arterial upgrade. Furthermore, the number of investigated patients in these studies (11, 13, 24–26) was small (1–21 patients), with high risk of bias, which might contribute to the wide range of survival outcomes. The registry of the Extracorporeal Life Support Organization (ELSO) showed a survival rate of 38% in patients requiring V-VA ECMO support (28). The reason for the more favorable outcome of patients in the current study might be explained by a more stringent selection of patients and by a homogenization focusing on a group with severe acute respiratory failure and a subsequent or concomitant cardio-circulatory deficit.

After hospital discharge, only 2 patients died during the 2-year follow-up, and survivors showed a surprisingly good organ function. Consistently, previous studies demonstrated good long-term outcomes after classical ECMO support (i.e., V-V- or V-A-cannulation) with most patients’ health almost restored to their previous level (1, 29, 30). V-A ECMO patients seem to have a worse long-term health status, what might be explained by a more serious initial clinical condition [e.g., acute (on chronic) heart failure, eCPR] (29). The fact that long-term outcome presented in this study is comparable with outcome of individuals after classical V-A or V-V EMCO support (1, 29, 30) shows that V-VA ECMO upgrading is feasible and should be considered for appropriate patients.

Identifying patients who will benefit from V-VA ECMO upgrade remains a challenge and poor selection is associated with unfavorable outcomes on ECMO (31, 32). We showed that the outcome of V-VA patients significantly worsened when their SOFA score exceeded 14 at the time of V-VA consideration. While the SOFA score was initially developed to describe the degree of organ function (18), it is increasingly used to predict mortality for patients with various conditions in the ICU (33, 34). While reliable data for triple cannulated V-VA ECMO patients are missing, a recent study found a higher SOFA score in non-survivors compared to survivors before classical V-V ECMO implantation, but with only moderate prognostic performance (35). In contrast, there was no difference in V-A ECMO patients in terms of survival reported in the same study (35). Besides the prognostic value of the SOFA-score as a global marker of organ dysfunction, we found that parameters of hemodynamic compromise, e.g., high serum lactate levels and an increased need for vasopressors, were associated with ICU-mortality. The high doses of vasopressors probably represent refractory circulatory failure with subsequent right ventricular dysfunction in a substantial proportion of patients (64%). After 24 h under V-VA ECMO support, vasopressor requirements were significantly lower indicating that V-VA ECMO could be effective in restoring hemodynamic stability in these patients.

While the significance of elevated serum lactate on outcome of patients who are commenced on either V-A- or V-V ECMO support is widely appreciated (36–38), this is to our knowledge the first study that extents the value of these clinical parameters to prognosis prediction before V-VA-cannulation. We found that elevated lactate levels independently predicted ICU-mortality. Therefore, the clinical decision for V-VA ECMO implementation should not rely solely on a risk score such as SOFA but be incorporated in the complex interaction of clinical status including lactate levels, need for vasopressor support and assessment of renal function in addition to clinical experience.

When patients develop cardio-circulatory failure while under V-V ECMO support, an alternative to V-VA upgrade might be converting from V-V to V-A cannulation. Falk et al. have shown that patients who required a conversion from V-V to V-A ECMO had a higher mortality than patients with initial V-A cannulation (39). Similarly, another study showed that initial V-A cannulation in ARDS patients is an independent predictor for increased mortality (40). In pronounced RV failure, adding a second venous drainage cannula (VV-A) to improve RV preload reduction and intracardiac shunt flow may be beneficial, but larger clinical studies have not been conducted to verify a clinical benefit.

In the current work, patients were approximately half of their overall ECMO runtime on V-VA configuration, suggesting that the need for respiratory support outlives the requirement for cardio-circulatory support. Since V-A ECMO support increases the risk for bleeding (41), renal failure, vascular complications and the Harlequin syndrome (42), downgrading V-VA to V-V cannulation, when hemodynamic stability has reached, might improve outcome compared to continued V-A ECMO support. In line with this approach, Stöhr et al. showed a lower 30-day-mortality for ARDS patients with V-VA cannulation when compared to V-A or V-V ECMO support (11).

Limitations of the present study are the retrospective design including missing data on follow-up and of hemodynamic variables. Since echocardiography data were not available in about half of the patients, the exact cause of acute cardiocirculatory failure could not be exactly differentiated in those patients. Due to similar reasons, extended hemodynamic monitoring was mostly not installed at the time of V-VA ECMO upgrade and retrospective interpretation is difficult under V-V ECMO support. However, insertion of the third arterial cannula was often carried out in an absolute emergency setting therefore not allowing performance of in depth echocardiography imaging. On the other hand, in only six patients the follow-up time was less than two years, allowing a reasonable interpretation of long-term outcome. Hemodynamic parameters other than vasopressor support and lactate after V-VA ECMO upgrade were not analyzed, hence limiting conclusions about the direct effect of V-VA ECMO. Because physiologic parameters are difficult to interpret retrospectively, mortality was chosen as a more robust endpoint. The analysis of three high-volume centers data might provide real-world clinical experience to ECMO providers. The design and missing data, however, limits the possibility of objectifying the individual clinical decisions that led to V-VA ECMO upgrade/cannulation. In addition, mechanisms leading to cardiocirculatory deterioration may differ between patients with initial triple cannulation and patients on V-V ECMO that were upgraded later to V-VA. Furthermore, patients were recruited over a long time span of 13 years in which the therapy of ARDS and handling of ECMO support has evolved (43). Therefore, the population is likely highly heterogeneous covering patients over a long time span and with no prespecified ethiological/physiological inclusion criteria but only clinical. Regarding the organ specific outcomes, patients with worse outcomes might have been more likely to drop out of the follow up. A prospective evaluation or matched cohort of patients with and without later V-VA ECMO upgrade would overcome most of the limitations above, but is unlikely to be conducted in the near future because of the time-critical setting and relatively few affected patients.

## Conclusion

In summary, this work demonstrated in the currently largest cohort of V-VA ECMO patients coming from V-V ECMO due to initial ARDS that approximately every second patient survived until hospital discharge. This encouraging survival rate was preserved over a two-year period where only a minority suffered from relevant organ dysfunction. Thus, an arterial upgrade of V-V ECMO patients suffering from ARDS to V-VA ECMO should not be rendered as futile *per se*. In our cohort, a SOFA score > 14 and elevated lactate levels at the time of V-VA upgrade evaluation predicted unfavorable outcome.

## Data availability statement

The data analyzed in this study was subject to the following licenses/restrictions: Authors can confirm that all relevant data are included in the article and/or its Supplementary material. The corresponding author may provide specified analyses or fully de-identified parts of the dataset upon reasonable request. Requests to access these datasets should be directed to SD, sascha.david@usz.ch.

## Ethics statement

The study was approved by the institutional review boards at all sites (Ethikkommission Hannover Medical School: #9720 BO K 2021, 2021/04/21; Kantonale Ethikkommission Zürich: ZH 2021-01804, 2021/10/08; and Ethikkommission University Hospital Bonn: #488/21, 2021/05/07). Informed consent was waived by the regulatory body for all patients at both sites in Germany and for patient in Zurich before 2016 and later if death occurred before consent could be obtained. Consent has been obtained for all patients not falling under above conditions.

## Author contributions

RE, LW, BS, A-KR, PW-G, SD, CB, and KS conceived and designed the research project. RE, BS, LW, A-KR, RA, MM, and PW-G handled the data acquisition. RE, BS, DH, PW-G, SD, CB, and KS analyzed the data. RE, LW, A-KR, and RA wrote the first draft of the manuscript. All authors substantially contributed to the interpretation of the data, critically revised the draft, read and approved the final manuscript, has agreed both to be personally accountable for the author’s own contributions, and to ensure that questions related to the accuracy or integrity of any part of the work, even ones in which the author was not personally involved, are appropriately investigated, resolved, and the resolution documented in the literature.
