# Supplementary material for: Outcomes of patients with acute respiratory failure on veno-venous extracorporeal membrane oxygenation requiring additional circulatory support by veno-venoarterial extracorporeal membrane oxygenation
Source: Front Med (Lausanne). 2022 Sep 23;9:1000084. doi: 10.3389/fmed.2022.1000084 (PMC9539450; doi:10.3389/fmed.2022.1000084)
Supplement: Supplementary file 1 [file Data_Sheet_1.pdf]

Supplemental Digital Content

Outcomes of patients with acute respiratory failure on veno-venous extracorporeal membrane oxygenation (ECMO) requiring additional circulatory support by veno-venoarterial ECMO

Rolf Erlebach, Lennart C. Wild, Benjamin Seeliger, Ann-Kathrin Rath, Rea Andermatt, Daniel A. Hofmaenner, Jens-Christian Schewe, Christoph C. Ganter, Mattia Müller, Christian Putensen, Ruslan Natanov, Christian Kühn, Johann Bauersachs, Tobias Welte, Marius M Hoeper, Pedro D Wendel-Garcia, Sascha David, Christian Bode, Klaus Stahl. for the BonHanZA (Bonn-Hannover-Zurich-ARDS) study group

TABLE OF CONTENT

Table S1: Missing data of variables in Table 1: Patient characteristic 2

Table S2: Missing data of variables in Table 2: Clinical condition and organ support 3

Table S3: Missing data of variables in Table 3: ECMO configuration, setting and complications 4

Table S4: Missing data of Table 4: Outcome and follow-up 5

Table S5: Outcome stratified by initial V-VA ECMO implantation vs. initial V-V ECMO implantation with later V-VA upgrade 6

Figure S1: Survival function from V-VA ECMO cannulation stratified by renal replacement therapy 7

Table S6: Association of VIS and absolute VIS reduction under V-VA ECMO support with 60-day intensive care unit mortality (multivariate cox proportional-hazards model) 8

Figure S2: Survival function from V-VA ECMO cannulation stratified by reduction of Vasoactive-inotropic score after 24 hours. 9

Table S1: Missing data of variables in Table 1: Patient characteristic

Variable	% Missing
Age, years	0
Sex, female	0
Body-mass index, kg/m2	0
ARDS, primary	0
Resp-Score diagnosis	4
COVID-19	0
Preserve Score	36
Sepsis	0
Comorbidities	
Adipositas	0
COPD	0
Arterial hypertension	0
Coronary artery disease	0
Congestive heart failure	0
Diabetes mellitus	0
Chronic kidney disease	0
Immunosuppression	0
Solid organ transplantation	0

Percentage of missing data is given for each variable. ARDS: acute respiratory distress syndrome, COPD: chronic obstructive pulmonary disease, COVID-19: Coronavirus disease 2019.

Table S2: Missing data of variables in Table 2: Clinical condition and organ support

Variables	% Missing V-V ECMO	% Missing V-VA ECMO
CPR before V-VA ECMO		0
Hospital admission to cannulation, days	0	0
ICU admission to cannulation, days	0	0
iMV to cannulation, days	0	0
SOFA score	13	3
Respiratory support	2	0
PEEP, cmH_2O	17	10
Minute ventilation, L/min	32	25
Plateau pressure, cmH_2O	40	26
SaO_2, %	9	11
PaO_2/FIO_2, mmHg	8	8
$PaCO_2$, mmHg	9	11
pH	13	12
Lactate, mmol/L	13	14
Inhalative nitric oxide	6	5
Norepinephrine	6	1
Norepinephrine dose, μg/kg/min	15	5
Epinephrine	8	1
Epinephrine dose, μg/kg/min	8	5
Dobutamine	8	1
Dobutamine dose, μg/kg/min	8	3
Vasoactive-inotropic score	15	7
LVEF	53	49
RVEF	53	47
Renal replacement therapy (n)	2	0

Percentage of missing data is given for each variable. CPR: cardiopulmonary resuscitation, FiO_2: fraction of inspired oxygen, HFOT: high-flow oxygen therapy, ICU: intensive care unit, iMV: invasive mechanical ventilation, LVEF: left ventricular ejection fraction, NIV: non-invasive ventilation, $PaCO_2$: partial pressure of carbon dioxide, PaO_2: partial pressure of oxygen, PEEP: positive end-expiratory pressure, RVEF: right ventricular ejection fraction, SaO_2: arterial oxygen saturation, SOFA: Sequential Organ Failure Assessment

Table S3: Missing data of variables in Table 3: ECMO configuration, setting and complications

Variables	% Missing
**ECMO cannulation (N = 73)**	
Venous drainage site	0
Venous return site	0
Arterial return site	0
Antegrade leg perfusion cannula	0
**V-V ECMO settings (N = 53)**	
Pump speed, rpm	30
Blood flow, L/min	13
Sweep gas flow, L/min	17
FsO_2	13
**V-VA ECMO settings (N = 73)**	
Pump speed, rpm	21
Total blood flow, L/min	0
Arterial blood flow, L/min	15
Sweep gas flow, L/min	7
**Complications of V-VA ECMO therapy (N = 73)**	
Complications during insertion	0
Complications during insertion requiring surgery	0
≥4 red blood cell concentrates / 24 hours	0
Major intracranial hemorrhage	0
Minor intracranial hemorrhage	0
Thromboembolic events	0
Leg ischemia	0
Other complications	0

Percentage of missing data is given for each variable. FsO_2: Sweep gas inlet oxygen fraction. Major intracranial hemorrhage: requiring neurosurgical intervention or resulting in any neurological deficit. Minor intracranial hemorrhage: occasionally identified on cerebral imaging. rpm: revolutions per minute.

Table S4: Missing data of Table 4: Outcome and follow-up

Variables	% Missing
ECMO runtime, days	1
V-VA ECMO runtime, days	0
ICU length of stay, days	4
Hospital length of stay, days	4
ICU mortality	0
Hospital mortality	0
Lung Transplantation	0
Mortality at 1 year	7
Mortality at 2 years	8
**Organ specific outcome at 2 years (N = 28)**	
Long-term oxygen therapy	7
Chronic kidney disease	7
Congestive heart failure	25

Percentage of missing data is given for each variable. ICU: intensive care unit, KDIGO: Kidney Disease: Improving Global Outcomes, NYHA: New York Heart Association

Table S5: Outcome stratified by initial V-VA ECMO implantation vs. initial V-V ECMO implantation with later V-VA upgrade

Variables	Initial V-VA (N=20)	Initial V-V with upgrade to V-VA (N=53)	p-value
ECMO runtime, days	12 (8-20)	12 (6-22)	0.673
V-VA ECMO runtime, days	6 (5-9)	6 (2-8)	0.244
ICU length of stay, days	34 (23-46)	30 (16-44)	0.282
Hospital length of stay, days	53 (34-86)	37 (17-72)	0.073
ICU mortality	8 (40)	27 (51)	0.567
Hospital mortality	9 (45)	28 (53)	0.738
Mortality at 1 year	9 (50)	30 (60)	0.647
Mortality at 2 years	9 (53)	30 (60)	0.822

Values are expressed as n (%) or median (interquartile range). ICU: intensive care unit.

Figure S1: Survival function from V-VA ECMO cannulation stratified by renal replacement therapy

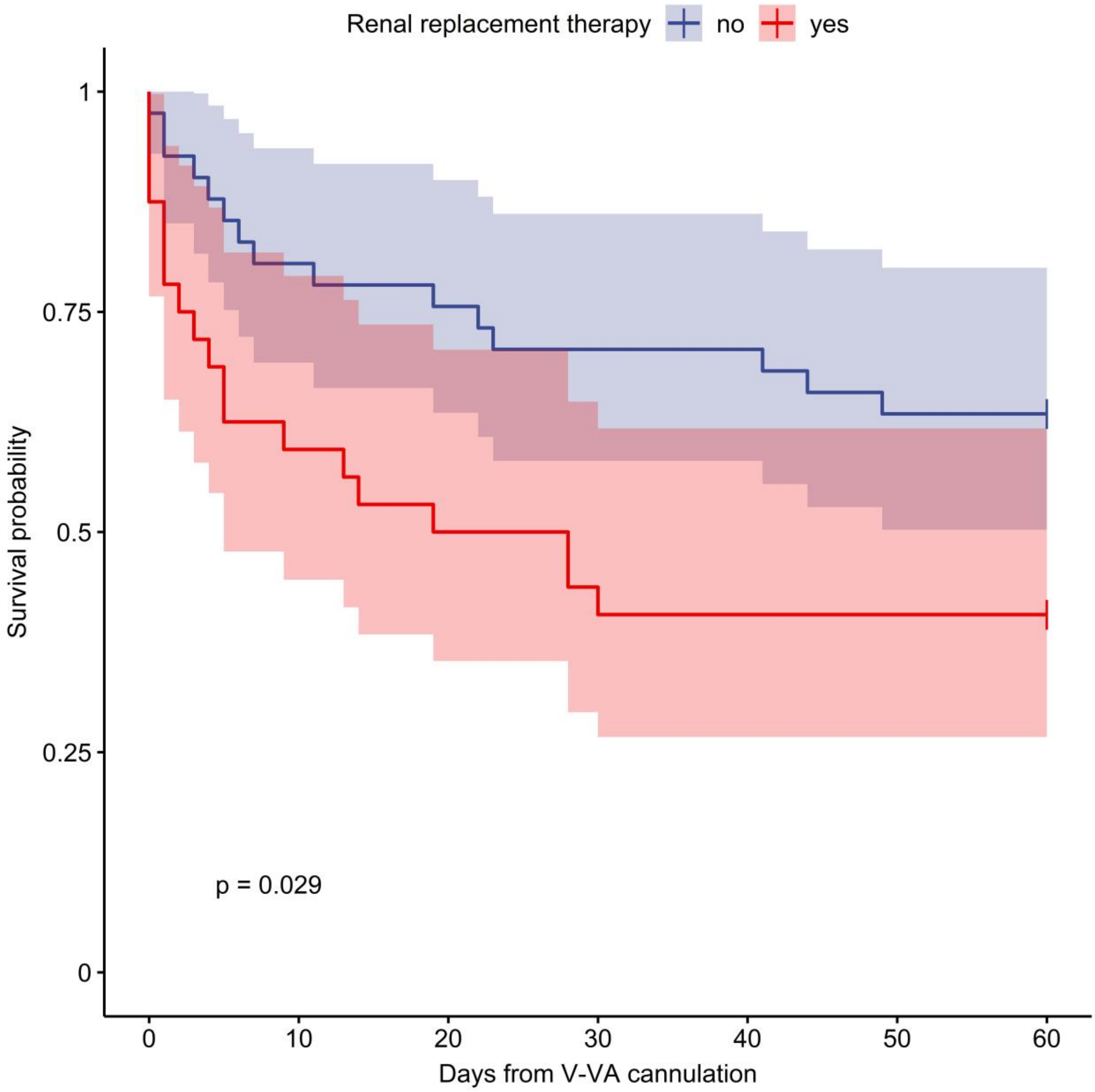

Table S6: Association of VIS and absolute VIS reduction under V-VA ECMO support with 60-day intensive care unit mortality (multivariate cox proportional-hazards model)

Variables	HR	CI 95%	p-value
VIS_{pre}	1.007	1.003-1.012	$p<0.001$
ΔVIS	0.995	0.993-0.998	$p<0.001$

Multivariate cox proportional-hazards model for 60-day intensive care unit-mortality. Values are expressed as Hazard ratio (HR) with 95% confidence interval (CI) and p-value. VIS_{pre}: VIS before V-VA ECMO upgrade, ΔVIS: absolute VIS reduction (VIS_{pre} – $VIS_{post24h}$), $VIS_{post24h}$: VIS 24 hours after V-VA ECMO upgrade, VIS: Vasoactive-inotropic score.

Figure S2: Survival function from V-VA ECMO cannulation stratified by reduction of Vasoactive-inotropic score after 24 hours.

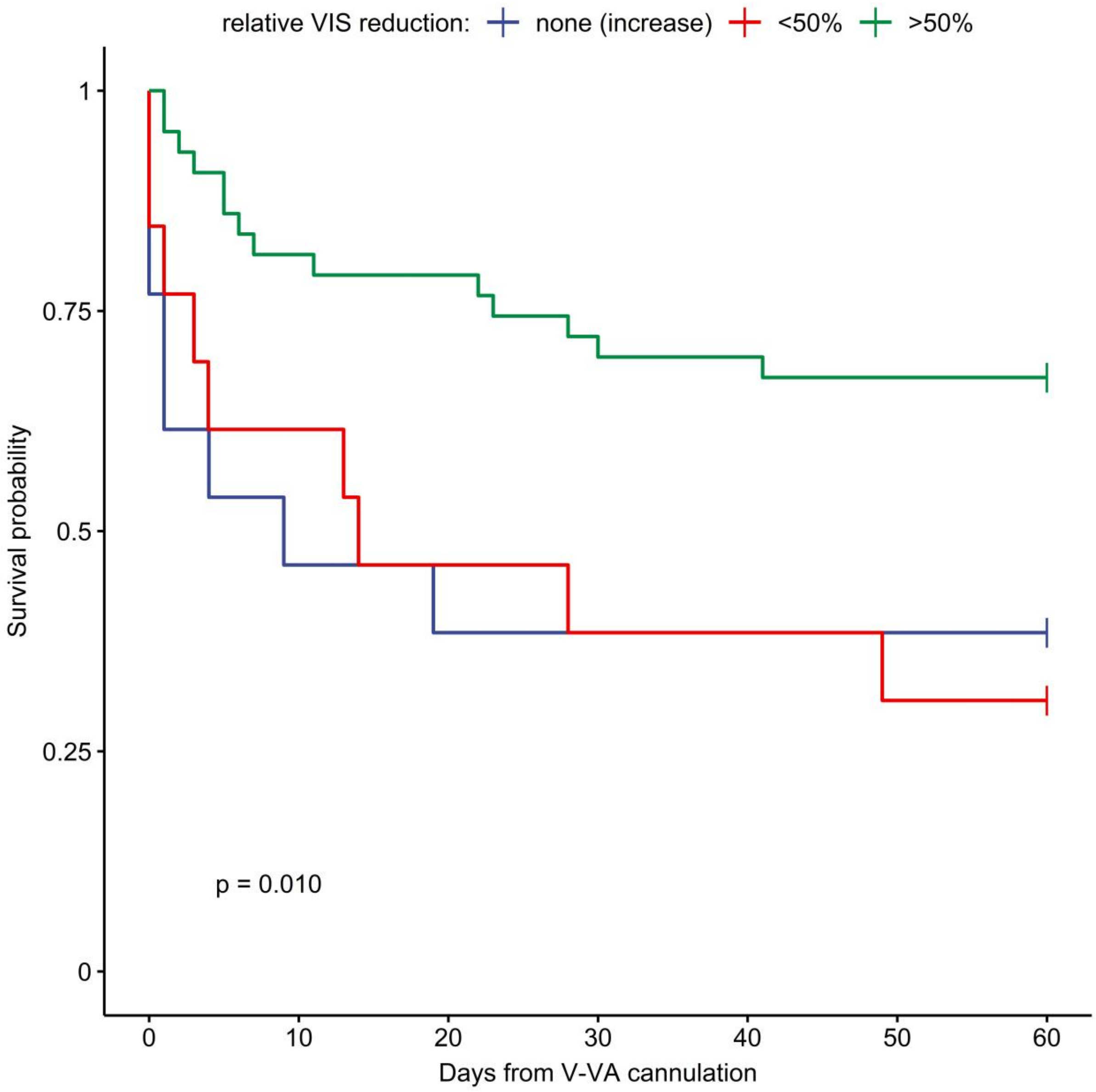

Relative reduction in Vasoactive-inotropic score (VIS) was calculated as $\frac{VIS_{pre} - VIS_{post24h}}{VIS_{pre}}$ (VIS_{pre} = before V-VA ECMO upgrade and $VIS_{post24h}$ = 24 hours after V-VA ECMO upgrade). If patients deceased in the first 24 hours after V-VA ECMO upgrade, the latest value before discontinuation of life-sustaining therapies was used (n = 5).
